# The impact of health literacy on treatment adherence in COPD patients: chain mediation roles of self-efficacy and perceived disease threat

**DOI:** 10.3389/fpubh.2025.1741304

**Published:** 2026-01-07

**Authors:** Fang Wang, Yun Qian, Limin Tian, Yaoyao Cui

**Affiliations:** Department of Respiratory and Critical Care Medicine, Beijing Genertec Aerospace Hospital, Beijing, China

**Keywords:** COPD patients, health literacy, perceived disease threat, self-efficacy, treatment adherence

## Abstract

**Background:**

Treatment adherence in patients with chronic obstructive pulmonary disease (COPD) is a critical factor influencing disease prognosis and quality of life. Health literacy, defined as patients’ ability to acquire and apply health information, may indirectly modulate adherence behaviors through psychological mechanisms. However, prior research has seldom examined the relationship between health literacy and treatment adherence, along with its underlying mechanisms. To bridge this gap, the present study, grounded in protection motivation theory and self-efficacy theory, investigates the mediating roles of perceived disease threat and self-efficacy in the association between health literacy and treatment adherence among COPD patients.

**Method:**

This study utilized a cross-sectional design and employed convenience sampling to recruit 456 COPD patients from four tertiary Grade A hospitals in Beijing, China. All participants completed the Health Literacy Scale, Perceived Disease Threat Scale, Self-Efficacy Scale, Treatment Adherence Scale, and provided essential demographic information.

**Result:**

Health literacy showed a significant positive correlation with treatment adherence, whereas perceived disease threat exhibited a significant negative correlation with self-efficacy. Perceived disease threat and self-efficacy demonstrated significant chain mediation in the relationship between health literacy and treatment adherence. Specifically, higher health literacy enhances patients’ confidence in implementing treatment regimens by fostering an understanding of treatment efficacy, enabling accurate perceptions of disease severity and personal susceptibility. This cognition translates into motivational urgency for action, thereby driving patients to engage in long-term and proactive adherence to various treatment behaviors.

**Conclusion:**

This study confirms that health literacy promotes treatment adherence in COPD patients via a chain mediation mechanism involving enhanced self-efficacy and reduced perceived disease threat. These findings offer a foundation for clinical interventions, such as elevating literacy through health education to optimize psychological pathways and improve patient management.

## 1 Introduction

Chronic obstructive pulmonary disease (COPD) is a common, preventable, and treatable chronic airway disorder (1, 2), characterized by persistent respiratory symptoms and airflow limitation resulting from airway and alveolar abnormalities (3, 4). As the third leading cause of death globally (5, 6), COPD affects approximately 384 million individuals (7), imposing substantial economic and societal costs through frequent hospitalizations, reduced productivity, and diminished quality of life (8, 9). Notably, COPD is an irreversible progressive disease (10), yet its course can be effectively managed and slowed through standardized pharmacotherapy, long-term oxygen therapy, pulmonary rehabilitation exercises, and lifestyle modifications (11, 12). However, research indicates that only about 30–60% of COPD patients maintain long-term adherence, leading to disease exacerbation, increased rehospitalization rates, and elevated healthcare costs, underscoring the relatively low treatment adherence in this population (13, 14). Treatment adherence, a core component of disease management, refers to the extent to which patients follow medical recommendations, encompassing medication use, lifestyle adjustments, and regular follow-ups (15, 16). It directly impacts disease control, exacerbation frequency, and long-term prognosis (17). Therefore, exploring and understanding key factors influencing treatment adherence in COPD patients is essential for optimizing prognosis, enhancing quality of life, and reducing disease burden.

The factors affecting treatment adherence in COPD patients are multifaceted, encompassing the complexity of treatment regimens, medication side effects, and variations in healthcare support, as well as deeper psychosocial influences (18, 19). Among these, patients’ health literacy has recently emerged as a pivotal variable impacting adherence and prognosis in chronic disease populations (20, 21). Health literacy represents patients’ capacity to obtain, comprehend, and apply health information to make informed decisions (22, 23). It encompasses functional, interactive, and critical dimensions, enabling patients to better grasp disease knowledge, evaluate treatment options, and execute self-management behaviors (24, 25). In chronic disease management, health literacy not only influences the extent of disease-related knowledge acquisition but also determines its translation into actionable health behaviors (26, 27). Existing studies suggest that patients with higher health literacy are better equipped to understand the importance of medication use, assess symptom risks, and proactively seek medical resources, thereby improving adherence (28, 29). However, current research on COPD cohorts has largely focused on the independent effects of health literacy and treatment adherence, with limited analysis of their interrelationship and even fewer explorations of underlying mechanisms. This gap hinders effective clinical interventions, lacking specificity and theoretical grounding. Thus, the present study aims to address the following questions: (1) Does health literacy in COPD patients exhibit a significant positive association with treatment adherence? (2) What are the internal mechanisms linking health literacy and treatment adherence in COPD?

Although public health systems have long assumed that increasing health literacy can facilitate positive behavioral change—an assumption that underlies many national Information, Education and Communication (IEC) strategies—the existing evidence base remains incomplete in several ways. IEC initiatives emphasize the dissemination of knowledge to enhance individuals’ understanding of disease risk and prevention, with the expectation that such cognitive gains will translate into improved adherence behaviors (30). However, most IEC frameworks conceptualize this process as a largely linear pathway from information acquisition to behavioral compliance. In COPD populations, where disease management is complex and behaviors are influenced by chronic symptoms, psychological burden, and long-term self-regulation demands, this assumption may be insufficient to explain actual adherence patterns. Critical questions remain regarding the specific psychological mechanisms through which health literacy exerts its influence, and why some individuals with adequate knowledge still exhibit suboptimal adherence. These gaps underscore the need for rigorous empirical testing of multi-stage mediation processes to inform the refinement of existing IEC-based public health strategies.

Self-efficacy refers to an individual’s belief in their ability to successfully execute required behaviors in specific situations to achieve desired outcomes (31). It is a dynamic cognitive process shaped by sources such as past experiences, vicarious learning, social persuasion, and physiological and emotional states (32, 33). Prior research confirms that COPD patients often confront symptoms like dyspnea, fatigue, and lifestyle changes (34), and those with high self-efficacy possess stronger beliefs in their capacity to persist with pharmacotherapy and sustained breathing exercises, effectively enhancing treatment adherence and promoting long-term persistence by buffering stress (35). Conversely, low self-efficacy may foster doubts about treatment regimens, leading to abandonment or irregular implementation of medical advice, accelerating disease progression and wasting healthcare resources (36, 37). Therefore, elevating patients’ self-efficacy is particularly crucial. According to self-efficacy theory, health literacy can bolster self-efficacy by enhancing knowledge reserves and decision-making confidence (38). COPD patients with high health literacy are more adept at interpreting medical information, assessing risks, and formulating feasible plans, further reinforcing their efficacy beliefs in self-management behaviors (39). For instance, when patients comprehend the harms of smoking on lung function and master cessation techniques, their self-efficacy increases, translating into higher adherence, such as regular inhaler use or follow-up participation. In contrast, low health literacy may lead to information misinterpretation, undermining self-efficacy and subsequently reducing adherence.

Perceived disease threat, a core element of protection motivation theory, includes perceived severity (an individual’s assessment of the potential consequences of the disease) and perceived susceptibility (cognition of personal risk for onset or worsening) (40). In prior research, numerous scholars have discussed how perceived disease threat enhances health behaviors (41, 42); for example, He et al. (43) reported that disease threats significantly reduce the purchase intention of ultra-processed foods among high-pressure workers, suggesting that heightened perceived threat can motivate individuals to avoid risky health behaviors. Zheng et al. (44) indicated that perceived disease threat in breast cancer patients and their spouses can directly or indirectly influence psychological distress through maladaptive or adaptive emotion regulation strategies. Although numerous studies have repeatedly confirmed that treatment adherence is a critical determinant of improved clinical outcomes, adherence remains consistently low across both chronic and acute illnesses (45). This has raised long-standing concerns regarding the underlying causes of poor adherence and the extent to which psychological and cognitive factors contribute to this problem (17). Among these factors, health literacy and perceived disease threat have been widely discussed in general chronic disease populations; however, their specific contributions to adherence deficits remain insufficiently quantified, particularly in COPD. Compared with other chronic conditions, COPD presents distinctive challenges—including progressive symptom fluctuation, complex inhalation techniques, long-term self-management demands, and heightened psychological burden—which may amplify the role of cognitive and emotional determinants (46, 47). Yet, very few studies have examined how much of COPD patients’ low adherence can be attributed to low health literacy and maladaptive risk perceptions, nor have they investigated the combined mediating effects of self-efficacy and perceived disease threat. Therefore, the present study focuses on COPD patients to address this critical evidence gap and to delineate the psychological mechanisms that may explain a substantial proportion of adherence behavior in this high-risk population.

In COPD, high perceived disease threat often stems from recurrent symptoms and prognostic uncertainty, exacerbating psychological burden (48, 49). However, moderate perceived disease threat can serve as a behavioral motivator, promoting adherence, such as encouraging regular check-ups and medication use. Prior research confirms that the facilitative role of perceived disease threat hinges on the moderating influence of health literacy (50, 51). Patients with low health literacy are prone to misinformation, amplifying perceived disease threat and leading to passive coping; those with high health literacy more accurately interpret symptom information and medical advice, forming moderate threat perceptions that avoid excessive fear or neglect, thereby motivating protective behaviors.

Based on the foregoing analysis, this study proposes a theoretical framework positing self-efficacy and perceived disease threat as chain mediators, elucidating how health literacy enhances self-efficacy, which in turn strengthens perceived disease threat, ultimately promoting treatment adherence. COPD patients with high health literacy are more inclined to accurately interpret medical information, thereby bolstering beliefs in their self-management capabilities and elevating confidence in addressing disease challenges. This augmented self-efficacy subsequently influences perceived disease threat; according to self-regulation models, high self-efficacy prompts more rational evaluations of disease severity and susceptibility, resulting in lower threat perception levels and stimulating protective behavior motivations. Given COPD’s progressive nature, which demands ongoing symptom monitoring, low health literacy often leads to insufficient self-efficacy, overestimation of disease threat, and diminished treatment adherence.

Drawing on the above analysis, this study proposes the following hypotheses:

*H1*: Health literacy in COPD patients has a significant positive effect on treatment adherence.

*H2*: Self-efficacy significantly mediates the relationship between health literacy and treatment adherence in COPD patients.

*H3*: Perceived disease threat significantly mediates the relationship between health literacy and treatment adherence in COPD patients.

*H4*: Self-efficacy and perceived disease threat exhibit significant chain mediation in the relationship between health literacy and treatment adherence in COPD patients.

## 2 Methods

### 2.1 Participants

#### 2.1.1 Ethical considerations

All procedures in this study adhered strictly to the ethical principles outlined in the Declaration of Helsinki. The study protocol was reviewed and approved by the Academic Ethics Committee of Beijing Genertec Aerospace Hospital (Approval no. 2025–0905-01). Researchers obtained written informed consent from all participants after fully explaining the study’s purpose, procedures, potential risks, and benefits, ensuring voluntary participation without coercion. To protect confidentiality, all data were anonymized.

#### 2.1.2 Study design

This study employed a cross-sectional observational design to investigate the relationships among health literacy, perceived disease threat, self-efficacy, and treatment adherence in patients with chronic obstructive pulmonary disease (COPD). Validated self-report scales were used to construct the study questionnaire, capturing associations among latent variables at a single time point. Structural equation modeling served as the primary analytical method to test the hypothesized mediation model, supplemented by bootstrapping to robustly infer indirect effects.

#### 2.1.3 Recruitment procedures

Researchers utilized a professional data collection platform^1^ to develop an electronic questionnaire. Participants were recruited from respiratory outpatient clinics and inpatient departments at four tertiary Grade A hospitals in Beijing between August and October 2025. A multi-step recruitment strategy was implemented to enhance sample representativeness and response rates. Specifically, collaborating pulmonologists identified eligible patients via electronic medical records, generating an initial list based on diagnostic criteria. Subsequently, researchers approached potential participants during routine outpatient visits, explaining the study’s purpose and content. Willing participants then completed the measurement items.

#### 2.1.4 Inclusion and exclusion criteria

This study established rigorous inclusion criteria. Participants were required to: (1) have a confirmed diagnosis of COPD according to the Global Initiative for Chronic Obstructive Lung Disease (GOLD) guidelines, verified by post-bronchodilator spirometry (FEV1/FVC < 0.70) (52, 53); (2) be aged 40 years or older, reflecting the typical onset of COPD; (3) possess sufficient cognitive and linguistic abilities to comprehend and complete the questionnaire, assessed by a Mini-Mental State Examination (MMSE) score ≥ 24 (54); (4) have received standard COPD treatment (e.g., bronchodilators, inhaled corticosteroids) for at least 6 months prior to enrollment (55, 56).

Exclusion criteria were applied to minimize confounding factors and ensure data validity: (1) acute exacerbation within the past 4 weeks, as this could distort reports of threat perception and adherence; (2) comorbid psychiatric disorders diagnosed via DSM-5 criteria that might impair self-efficacy assessment (e.g., severe depression or anxiety) (57); (3) history of substance abuse or dependence, which could affect health literacy; (4) inability to read or write in Chinese; (5) concurrent participation in other interventional studies that might alter treatment adherence behaviors; (6) incomplete questionnaires or responses exhibiting strong response consistency biases.

#### 2.1.5 Minimum sample size

The minimum sample size was determined *a priori* using G*Power software (Version 3.1) to ensure adequate statistical power for detecting mediation effects in structural equation modeling. Based on prior literature indicating a medium effect size for the association between health literacy and adherence (Cohen’s f^2^ = 0.15), and assuming a chain mediation pathway with two mediators, we calculated the required sample for multiple regression analysis with up to 12 predictors (including covariates), targeting 80% power at an alpha level of 0.05. This yielded a minimum recruitment target of 127 participants.

#### 2.1.6 Study sample

A total of 481 participants meeting the inclusion and exclusion criteria were recruited. During data cleaning, 19 incomplete questionnaires and 6 exhibiting strong response consistency were excluded. The final effective sample comprised 456 COPD patients, with a validity rate of 95.16%. Among them, 260 (57.0%) were male and 196 (43.0%) were female. Disease severity distribution was as follows: 31.6% in GOLD stages I/II (mild/moderate), 41.7% in stage III (severe), and 26.8% in stage IV (very severe). Educational attainment varied, with 50% having primary/junior high school education, 27.4% having junior high to senior high school education, and 22.6% having university education or higher. Detailed demographic information is presented in Table 1.

**Table 1:** Demographic characteristics of COPD patients.

Variables	Items	Frequency (n)	Percentage (%)
Gender	Male	260	57.0%
	Female	196	43.0%
Age (year)	M ± SD	60.535 ± 12.529	
Educational level	Primary school and below	228	50.0%
	Junior high school to senior high school	125	27.4%
	Undergraduate degree and above	103	22.6%
Occupational status	Retirement	135	29.6%
	On the job	219	48.0%
	Freelance work	102	22.4%
Monthly income level	≤3,000	64	14.0%
	3,001–6,000	125	27.4%
	6,001–9,000	189	41.4%
	9,001 and above	78	17.1%
Smoking history	Yes.	271	59.4%
	No.	185	40.6%
Course of disease	<1 year	72	15.8%
	1–3 year	158	34.6%
	3–5 year	152	33.3%
	≥5 year	74	16.2%
GOLD classification	I/II	144	31.6%
	III	190	41.7%
	IV	122	26.8%

### 2.2 Study instruments

#### 2.2.1 Health literacy scale

Health literacy was measured using the 14-item Chinese version of the Health Literacy Scale developed by Wu et al. (58). This scale was originally designed for Chinese patients with type 2 diabetes and underwent rigorous translation and cross-cultural adaptation following Brislin’s model to ensure conceptual equivalence. It comprises 14 items across three dimensions: functional health literacy (5 items), assessing basic reading and comprehension of health-related materials; communicative health literacy (5 items), evaluating the ability to actively seek, understand, and communicate health information; and critical health literacy (4 items), measuring the capacity to critically evaluate and apply information for informed health decisions. Items were scored on a 5-point Likert scale ranging from “strongly disagree” to “strongly agree.” Total scores range from 14 to 70, with higher scores indicating greater health literacy. In this study, the scale demonstrated excellent internal consistency (Cronbach’s *α* = 0.921).

#### 2.2.2 Perceived disease threat scale

Perceived disease threat was assessed using the Brief Illness Perception Questionnaire (BIP-Q) developed by Broadbent et al. (59). This scale, grounded in the self-regulation model, is a widely used tool for evaluating individuals’ cognitive and emotional representations of illness. It consists of 8 items covering aspects such as consequences, timeline, personal control, treatment control, identity, concern, emotional response, and illness comprehensibility. The scale has been adapted into Chinese versions for various patient populations, including breast cancer patients (60), hypertensive groups (61), and stroke patients (62). In this study, it was employed to measure perceived disease threat in COPD patients, with items scored on a 5-point Likert scale (1 = strongly disagree, 5 = strongly agree). Total scores are calculated by summing items 1, 2, 5, 6, and 8, and reverse-scoring items 3, 4, and 7. Lower scores indicate a stronger perception of the illness as a threat. The scale showed excellent internal consistency in this study (Cronbach’s *α* = 0.942).

#### 2.2.3 Self-efficacy scale

Self-efficacy was measured using the General Self-Efficacy Scale developed by Chen et al. (63). This 8-item, single-dimension instrument includes statements such as “I will be able to achieve most of the goals I have set for myself.” It has been widely applied among Chinese chronic disease patients (50, 64) and adapted into a Chinese version by Wang et al. (65). Items were scored on a 5-point Likert scale from 1 (strongly disagree) to 5 (strongly agree), with total scores ranging from 8 to 40. Higher scores reflect stronger self-efficacy. The scale exhibited excellent internal consistency in this study (Cronbach’s *α* = 0.937).

#### 2.2.4 Treatment adherence scale

Treatment adherence was evaluated using the Adherence in Chronic Diseases Scale (ACDS) developed by Kosobucka et al. (66). This 7-item scale employs a 5-point Likert scale for scoring (1 = strongly disagree, 5 = strongly agree), with total scores ranging from 7 to 35. Items 1–5 assess medication behaviors, while items 6–7 address the physician-patient relationship, which indirectly influences adherence. Adherence levels are categorized based on total scores: high (>33), medium (28–33), and low (<28). The scale has been extensively used in chronic disease populations (74, 75) to quantify adherence and identify potential barriers to treatment implementation. Thus, it was adopted here to assess medication adherence in COPD patients. The scale demonstrated good internal consistency in this study (Cronbach’s *α* = 0.876).

### 2.3 Statistical analysis

All statistical analyses were conducted using IBM SPSS Statistics (Version 27.0). First, common method bias was examined to assess potential biases in the study. Descriptive statistics were then used to evaluate the distribution of variables. Next, normality assumptions were assessed via skewness and kurtosis values; skewness < |3| and kurtosis < |8| indicated acceptable univariate normality for structural equation modeling (67). Subsequently, correlation analyses were performed to examine inter-variable relationships and their strengths. Third, SPSS macro Process Model 6 was utilized to test the chain mediation model involving perceived disease threat and self-efficacy, with health literacy as the independent variable, perceived disease threat and self-efficacy as chain mediators, and treatment adherence as the dependent variable. This model incorporated direct paths from health literacy to treatment adherence, as well as indirect paths through the chain mediators, while controlling for covariates to isolate psychosocial mechanisms. Model fit was evaluated using multiple indices: chi-square (χ^2^) statistic, comparative fit index (CFI > 0.90), Tucker-Lewis index (TLI > 0.90), root mean square error of approximation (RMSEA < 0.08), and others. Bias-corrected bootstrapping with 5,000 resamples was employed to estimate indirect effects, generating 95% confidence intervals (CIs) to provide robust inferences for mediation without relying on normality assumptions.

## 3 Results

### 3.1 Common method bias test

Given that all measurement scales in this study employed a 5-point Likert format, with data sourced from the same location and potentially influenced by social desirability, common method bias may be present. Therefore, we safeguarded respondents’ anonymity and data confidentiality to mitigate such bias.

We conducted Harman’s single-factor test by subjecting all variable items to exploratory factor analysis. The results indicated that the first factor accounted for 17.781% of the total variance, below the critical threshold of 40%. Thus, common method bias was not a concern in this study.

### 3.2 Descriptive statistics and correlation analysis

Descriptive statistics and correlation analyses were performed for health literacy, perceived disease threat, self-efficacy, and treatment adherence, as shown in Tables 2, 3. Among COPD patients, the mean score for health literacy was 3.384 (SD = 0.601), for perceived disease threat was 2.530 (SD = 0.637), for self-efficacy was 3.435 (SD = 0.607), and for treatment adherence was 3.442 (SD = 0.641). The mean scores for these core variables all exceeded the midpoint (M = 2.5) of the 5-point Likert scale. Skewness values ranged from −0.816 to 0.610, and kurtosis values ranged from 0.845 to 2.003. According to Kline (67) criteria for normality, the core variables approximated a normal distribution.

**Table 2:** Descriptive statistics for core variables.

Variables	*M*	SD	Skewness	Kurtosis
Health literacy	3.384	0.601	−0.816	2.003
Self-efficacy	3.435	0.607	−0.739	1.555
Perceived disease threat	2.530	0.637	0.610	1.165
Treatment adherence	3.442	0.641	−0.608	0.845

**Table 3:** Correlation analysis for core variables.

Variables	1	2	3	4
Health literacy	1			
Self-efficacy	0.576**	1		
Perceived disease threat	−0.512**	−0.578**	1	
Treatment adherence	0.510**	0.481**	−0.556**	1

**p < 0.01.

Correlation analyses revealed a significant negative correlation between health literacy and perceived disease threat (*r* = −0.512, *p* < 0.01); a significant positive correlation between health literacy and self-efficacy (*r* = 0.576, p < 0.01); and a significant positive correlation between health literacy and treatment adherence (*r* = 0.510, *p* < 0.01). Self-efficacy showed a significant negative correlation with perceived disease threat (*r* = −0.578, *p* < 0.01) and a significant positive correlation with treatment adherence (*r* = 0.481, *p* < 0.01). Perceived disease threat exhibited a significant negative correlation with treatment adherence (*r* = −0.556, *p* < 0.01).

### 3.3 Chain mediation analysis of perceived disease threat and self-efficacy

Health literacy was treated as the independent variable, perceived disease threat and self-efficacy as mediators, and treatment adherence as the dependent variable. We constructed a structural equation model using AMOS 29.0 to test the research hypotheses. Confirmatory factor analysis demonstrated excellent model fit: *χ*^2^/df = 1.183; CFI = 0.945; GFI = 0.922; AGFI = 0.912; TLI = 0.941; RMSEA = 0.020.

The results indicated that health literacy exerted a significant negative direct effect on perceived disease threat [*β* = −0.283, *p* < 0.001, 95% CI = (−0.377, −0.189)]; health literacy had a positive direct effect on self-efficacy [*β* = 0.582, *p* < 0.001, 95% CI = (0.506, 0.658)]. Self-efficacy negatively predicted perceived disease threat [*β* = −0.445, *p* < 0.001, 95% CI = (−0.538, −0.352)]; perceived disease threat negatively predicted treatment adherence [*β* = −0.352, *p* < 0.001, 95% CI = (−0.445, −0.259)]. Self-efficacy positively predicted treatment adherence [*β* = 0.139, *p* < 0.01, 95% CI = (0.037, 0.241)]. Health literacy had a significant positive effect on treatment adherence [*β* = 0.273, *p* < 0.001, 95% CI = (0.175, 0.371)]. Detailed results are presented in Table 4.

**Table 4:** Regression equation model analysis for self-efficacy and perceived disease threat.

Model	Dependent variable	Independent variable	*R*	*R* ^2^	*F*	*β*	t	LLCI	ULCI
Model 1	Self-efficacy	Health literacy	0.576	0.332	225.366***	0.582	15.012***	0.506	0.658
Model 2	Perceived disease threat	Health literacy	0.618	0.382	140.041***	−0.283	−5.914***	−0.377	−0.189
		Self-efficacy				−0.445	−9.392***	−0.538	−0.352
Model 3	Treatment adherence	Health literacy	0.623	0.388	95.636***	0.273	5.473***	0.175	0.371
		Self-efficacy				0.139	2.676**	0.037	0.241
		Perceived disease threat				−0.352	−7.470***	−0.445	−0.259

***p < 0.001; **p < 0.01.

**Figure 1:**
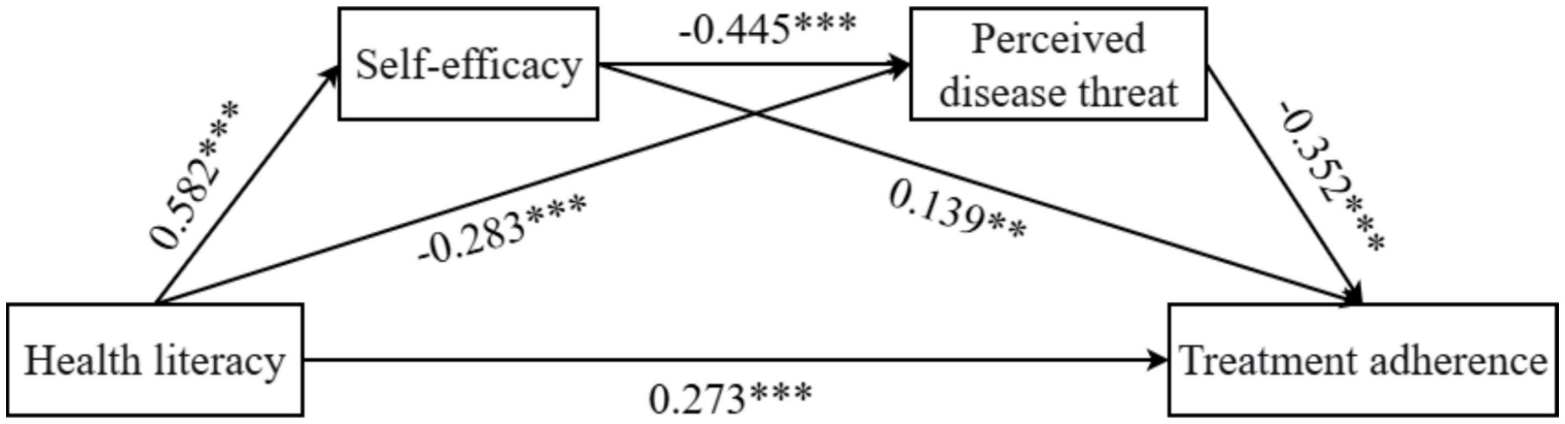
Graph of path coefficients for chain mediation, ***p < 0.001; **p < 0.01.

Mediation effect analysis, as shown in Table 5 and Figure 1, revealed that the total effect of health literacy on treatment adherence was 0.544, of which 49.9% was explained by the mediating roles of self-efficacy and perceived disease threat. Further examination showed a significant chain mediation effect via health literacy → self-efficacy → perceived disease threat → treatment adherence [*β* = 0.091, SE = 0.022, 95% CI = (0.051, 0.137)], accounting for 16.7% of the total effect. For individual mediation paths, health literacy → self-efficacy → treatment adherence exhibited a significant mediating effect [*β* = 0.081, SE = 0.038, 95% CI = (0.008, 0.157)], representing 14.9% of the total effect; health literacy → perceived disease threat → treatment adherence showed a significant mediating effect [*β* = 0.100, SE = 0.025, 95% CI = (0.056, 0.150)], comprising 18.3% of the total effect.

**Table 5:** Effect decomposition for chain mediation.

Effect	*β*	SE	LLCI	ULCI	Effect proportion	Supporting hypothesis
Total effect	0.544	0.043	0.460	0.629	100%	
Direct effect	0.273	0.050	0.175	0.371	50.1%	H1
Total	0.272	0.047	0.181	0.364	49.9%	
Ind1	0.081	0.038	0.008	0.157	14.9%	H2
Ind2	0.100	0.025	0.056	0.150	18.3%	H3
Ind3	0.091	0.022	0.051	0.137	16.7%	H4

Ind1:health literacy → self-efficacy → treatment adherence; Ind2: health literacy → perceived disease threat → treatment adherence; Ind3: health literacy → self-efficacy → perceived disease threat → treatment adherence.

## 4 Discussion

### 4.1 Theoretical implications

This study empirically demonstrates the interrelationships among health literacy, perceived disease threat, self-efficacy, and treatment adherence in patients with chronic obstructive pulmonary disease (COPD). The findings confirm that health literacy indirectly promotes treatment adherence through a chain mediation mechanism involving self-efficacy and perceived disease threat. This revelation elucidates the underlying psychological mechanisms by which health literacy influences patient adherence behaviors, bridging the theoretical gap in life behavior intervention research from knowledge acquisition to actionable cognition. The results align with the core assumptions of the health belief model and protection motivation theory, while extending their applicability, particularly in the domain of respiratory chronic disease management, thereby providing a novel theoretical framework for explaining patient treatment behaviors.

This study supports a significant positive association between health literacy and treatment adherence, further unveiling the mediating roles of perceived disease threat and self-efficacy. Grounded in the health belief model, individual behavioral motivation stems from perceptions of disease risk and beliefs in one’s behavioral efficacy (68), with health literacy serving as a cognitive enhancer. Further analysis reveals that high levels of health literacy not only assist patients in accurately comprehending disease severity but also prevent excessive fear or cognitive distortions, fostering moderate perceived disease threat and thereby bolstering self-efficacy to elicit positive treatment behaviors. Conversely, patients with low health literacy may amplify disease threats due to misinformation, negative beliefs, or barriers in patient-provider communication, undermining self-management beliefs and leading to reduced adherence. This partially explains the paradox observed in prior research of high knowledge yet low adherence.

The study findings further corroborate the mediating role of perceived disease threat in the link between health literacy and adherence. Diverging from traditional views treating it as a singular variable, this research identifies perceived disease threat as a pivotal psychological node in converting cognitive signals into actions, achieving behavioral internalization through the activation of self-efficacy. Specifically, elevated health literacy enables patients to accurately evaluate disease consequences and controllability, transforming external compliance with treatment into internal endorsement. Self-efficacy acts as a bridge in this process, converting rational cognition into the psychological drive for sustained action (69). This model validates the dynamic interplay among environmental cognition, individual beliefs, and behavioral outcomes in social cognitive theory, while enriching the motivational composition framework of protection motivation theory—namely, that individuals can stabilize protective behaviors through confidence-building only after fully understanding threats and resources. The model’s robust stability and fit indices further enhance the credibility and external validity of these results.

### 4.2 Practical and clinical implications

This study reveals that health literacy not only directly influences treatment adherence in COPD patients but also indirectly fosters sustained treatment behaviors through a chain mediation pathway involving self-efficacy and perceived disease threat. These findings provide robust empirical support and theoretical foundations for redesigning psychological interventions and health education models in chronic disease management. The core contribution lies in systematically validating, for the first time in human chronic disease psychobehavioral mechanisms, the dynamic transmission pathway of “cognitive resources—subjective beliefs—risk perception—behavioral decision-making,” thereby deepening the psychological connotations of health literacy and expanding the explanatory boundaries of protection motivation theory and social cognitive theory in clinical behavior domains.

First, from the perspective of cognitive-behavioral interventions, the results suggest that merely enhancing patients’ medical knowledge reserves is insufficient for behavioral transformation; rather, the processing of health information and the psychological belief system are the decisive factors for sustained adherence. Health literacy aids individuals in more accurately understanding treatment plans and risk information, thereby alleviating cognitive uncertainty, while self-efficacy translates this cognitive advantage into an “I can do it” behavioral motivation. Moreover, moderate perceived disease threat, under the synergistic regulation of individual cognitive resources and belief structures, can elicit rational defensive actions rather than anxiety-driven avoidance. Therefore, clinical interventions should concurrently address patients’ cognitive enlightenment, self-efficacy reinforcement, and risk perception modulation, promoting adherence internalization through psychological empowerment.

Second, at the level of medical practice and patient education, the model’s evidence-based support informs precision chronic disease management. Healthcare providers should incorporate multi-tiered health literacy intervention strategies during treatment: at the cognitive level, establish health literacy profiles based on stratified assessments, utilizing multimodal, accessible educational materials (e.g., interactive videos, visual diagrams, concise health guides) to improve information comprehension; at the belief level, enhance patient self-efficacy through individualized goal management, behavioral feedback, and peer modeling support, encouraging proactive participation in disease management within controllable scopes; at the emotional level, facilitate appropriate disease threat cognition via risk communication training, avoiding excessive fear or complacency to maintain vigilant yet rational health motivations.

Health literacy challenges are not confined to COPD populations or to a single country; rather, wide disparities in health literacy exist globally, with many regions reporting limited public awareness, inadequate comprehension of disease information, and substantial gaps in self-management capabilities (70). International studies have shown that low health literacy is common in both high-income and low- to middle-income countries, often contributing to delayed diagnosis, poor treatment adherence, and suboptimal chronic disease outcomes (71). These disparities highlight a pressing global need to strengthen health literacy through coordinated public health strategies, culturally adapted education materials, and improved health communication systems. The present study contributes to this global agenda by demonstrating how psychological mechanisms link health literacy to behavior, offering insights that may be applicable to diverse populations. By clarifying how self-efficacy and perceived disease threat influence adherence, our findings underscore the importance of developing globally relevant interventions that go beyond information dissemination and instead empower individuals to interpret, internalize, and act on health information in meaningful ways.

Traditional IEC materials often focus predominantly on transmitting factual information; however, our results suggest that such approaches may be insufficient unless they simultaneously reinforce patients’ confidence in managing treatment tasks and help them form accurate, moderate perceptions of disease threat. By integrating explicit self-efficacy enhancement strategies and calibrated risk communication into IEC programs, health systems can shift from knowledge provision alone toward psychologically informed interventions that more reliably induce adherence. This refined model offers a pathway to strengthen community-level disease management efforts and thereby supports the overarching goals of IEC: reducing disease burden through sustained behavioral engagement.

While IEC interventions were originally designed to enhance public awareness and promote treatment adherence through improved health literacy, their real-world effectiveness has remained limited. This is largely due to inadequate resource allocation, insufficiently trained communication personnel, and the lack of innovative, population-tailored strategies for building social awareness. Consequently, IEC functions are often underdeveloped across the continuum of care—from preventive and promotive services to curative, rehabilitative, and palliative care—and across primary, secondary, and tertiary levels of the health system. Our findings underscore the urgent need to redesign IEC components in a more evidence-based and psychologically informed manner. In addition to disseminating information, IEC initiatives must incorporate structured approaches to enhance patients’ self-efficacy and calibrate perceived disease threat, as these mechanisms play a critical role in transforming knowledge into behavior. Furthermore, the importance of health literacy for self-management is increasingly recognized in a wide range of non-communicable diseases, such as hypertension, diabetes, and cancer screening. Strengthening IEC capacity therefore requires expanding investment in trained health educators, developing engaging and culturally sensitive communication materials, and integrating behavioral science principles into public health messaging. Accordingly, the results of this study provide a theoretical and empirical foundation for policymakers to reinforce and modernize IEC strategies in order to improve long-term chronic disease management and reduce population-level disease burden.

Furthermore, the theoretical model holds significant implications for public health policy and chronic disease management system development. Health literacy can be regarded as a foundational health competency in national chronic disease prevention frameworks, warranting continuous enhancement through community health education, digital health platforms, and “clinician-patient-family” tripartite collaboration mechanisms. Integrating health literacy assessments into COPD stratified care systems can optimize patient risk stratification and individualized management, shifting paradigms from “medically oriented” to “patient cognition-oriented.” Additionally, reinforcing self-efficacy and optimizing threat perception can serve as intermediate psychological indicators in health promotion programs, offering novel operational pathways for integrating precision medicine with psychological health interventions.

### 4.3 Influence of healthcare financing structures on treatment adherence

When explaining the psychological mechanism in this study, it’s important to also consider another key external factor that can affect adherence behavior: the structure of medical cost payment. Our sample comes from top-tier hospitals in Beijing, where patients generally benefit from a well-developed public funding and health insurance system. As a result, economic burdens have less direct impact on treatment adherence. However, in many countries or regions where out-of-pocket payments dominate or drug co-pays are high, the financial cost itself becomes a critical determinant of adherence. Previous research has shown that even patients with high health literacy and clear awareness of disease risks may significantly reduce their adherence due to heavy medication expenses, making it difficult to sustain standardized treatment long-term (72, 73).

Therefore, the psychological mechanism model developed in this study is more applicable to contexts with solid healthcare coverage and relatively low patient cost-sharing. In countries or populations facing greater economic burdens, the behavioral benefits gained from improved health literacy may be partly offset by high expenses. Future research should incorporate systemic factors such as “medical cost burden,” “payment methods,” and “reimbursement rates” into expanded models and examine their interactions or moderating effects with psychological variables. This would help build a more comprehensive framework of treatment adherence determinants and enhance the cross-cultural generalizability of the findings.

### 4.4 Policy-oriented

While the present findings are derived from COPD patients, the mechanisms identified—linking health literacy to treatment adherence through self-efficacy and calibrated disease threat—offer broader policy implications that extend well beyond traditional IEC efforts. The results provide policymakers with a theoretically grounded framework for strengthening health literacy at the population level through more targeted and psychologically informed programmatic interventions.

A major challenge in public health is achieving high levels of health literacy across diverse communities using scalable and resource-sensitive strategies. Although resource constraints are well known, certain intervention models can substantially enhance the effectiveness of existing IEC systems without requiring major structural expansion. Three illustrative examples demonstrate how policy makers can translate the mechanisms identified in this study into actionable health literacy strategies.

#### 4.4.1 Clinical-embedded literacy enhancement programs

Healthcare providers can integrate brief, structured “teach-back” and “demonstration-based” education sessions into routine care, especially for chronic diseases requiring complex self-management. Evidence shows that even low-cost modules—such as nurse-led inhaler technique coaching or visual risk explanation charts—can increase self-efficacy, reduce exaggerated threat perceptions, and improve adherence. Embedding these modules into electronic medical records as required steps can ensure sustainability and standardization.

#### 4.4.2 Digital and mobile health literacy platforms

Policymakers can expand digital tools designed to provide simplified, interactive disease information, risk interpretation, and step-by-step behavioral guidance. Mobile apps, short video animations, interactive decision aids, and symptom-tracking dashboards can enhance functional and critical health literacy while also reinforcing self-efficacy by giving patients real-time feedback. Leveraging low-cost digital dissemination (e.g., community QR codes, social media micro-lessons) can compensate for human resource shortages in IEC units.

#### 4.4.3 Community-based literacy support and peer-led models

Community health workers and trained peer educators can deliver culturally tailored messages, group demonstrations, and behavior modeling sessions. Peer support groups—already used in diabetes and cancer self-management programs—can empower patients through shared experiences, thus improving perceived controllability and reducing debilitating threat perceptions. These models can be implemented through existing community health centers with minimal additional cost.

Collectively, these examples demonstrate how the psychological mechanisms identified in this study can be operationalized to enhance health literacy and adherence in real-world settings. By moving beyond information dissemination alone and incorporating strategies that target self-efficacy and adaptive risk perception, policymakers can upgrade current IEC practices and improve chronic disease outcomes across multiple levels of care.

### 4.5 Limitations and future directions

Although this study provides novel empirical evidence for understanding the psychological mechanisms of health literacy in COPD patients, several limitations and avenues for improvement remain. Given the cross-sectional design of this study, causal relationships among health literacy, self-efficacy, perceived disease threat, and treatment adherence cannot be inferred. The associations identified reflect correlational patterns at a single time point, and the design does not permit any directional or temporal conclusions. For instance, patients with high adherence may develop elevated health literacy and self-efficacy through long-term treatment experiences. Future research could employ longitudinal designs or experimental interventions to verify the dynamic effects of health literacy interventions on self-efficacy, perceived disease threat, and adherence over time, thereby strengthening causal explanations.

Second, the sample was drawn from patients at tertiary Grade A hospitals in Beijing, potentially exhibiting higher healthcare accessibility and cognitive levels, limiting generalizability to primary care or rural COPD populations. Subsequent studies should expand sample sources to include patients from diverse regions and cultural backgrounds, utilizing multicenter stratified sampling to enhance sample heterogeneity and the external validity of results.

All measurement instruments were self-report scales, susceptible to social desirability bias and common method variance. Although this study mitigated risks through anonymous responses and statistical tests, future research could incorporate objective behavioral data (e.g., medication usage records, follow-up attendance data, or wearable monitoring metrics) to validate adherence measurements, establishing composite models based on objective behavioral indicators.

Another limitation of this study is the absence of a control group. The inclusion of age- and gender-matched normal controls and diseased controls would have enabled direct comparison of health literacy levels across different population groups in Beijing. Such comparisons could help determine whether the observed patterns are unique to COPD patients or reflect broader population trends. Without these control groups, the generalizability of the findings—particularly regarding the relative impact of health literacy—remains restricted. Future research should incorporate matched control samples to strengthen comparative validity and to more precisely evaluate the specificity of health literacy and psychological mechanisms in COPD.

Finally, this study did not fully account for potential moderating variables. For example, social support, cultural orientations, and patient-provider trust may influence the formation pathways of self-efficacy and threat perception. Future work could build multilayer moderated mediation frameworks on the chain mediation model to explore the impact of socio-contextual variables on model stability. Additionally, potential interactive effects of demographic variables such as gender, age, education level, and disease duration should be considered to uncover psychological mechanism differences across subgroups.

## 5 Conclusion

Focusing on COPD patients and grounded in protection motivation theory and self-efficacy theory, this study systematically elucidates the mechanisms among health literacy, self-efficacy, perceived disease threat, and treatment adherence. The results indicate that health literacy not only directly promotes patient treatment adherence but also exerts significant chain mediation effects by enhancing self-efficacy and modulating perceived disease threat, constructing a psychological pathway model from “cognitive comprehension—belief reinforcement—behavior maintenance.” These findings not only affirm the central role of health literacy in chronic disease management but also deepen the understanding of the intrinsic psychological mechanisms underlying patient adherence behaviors, offering actionable intervention strategies for clinical practice. In the future management of COPD and other chronic diseases, comprehensive strategies combining stratified health education, psychological empowerment, and risk perception optimization should be implemented to facilitate patients’ transition from passive compliance to proactive treatment, thereby achieving dual enhancements in long-term adherence and quality of life.

## Data Availability

The raw data supporting the conclusions of this article will be made available by the authors, without undue reservation.

## Footnotes

1 Credamo, https://www.credamo.com/
